# [Retracted] LncRNA TTN‑AS1 promotes endometrial cancer by sponging miR‑376a‑3p

**DOI:** 10.3892/or.2024.8820

**Published:** 2024-10-07

**Authors:** Longde Shen, Yinyin Wu, Ailu Li, Lichun Li, Longyuan Shen, Qiuxia Jiang, Qiuxia Li, Zhifen Wu, Liji Yu, Xiaohong Zhang

Oncol Rep 44: 1343–1354, 2020; DOI: 10.3892/or.2020.7691

Following the publication of this paper, it was drawn to the Editor's attention by a concerned reader that certain of the Transwell cell migration and invasion assay data in Fig. 2E and F and 4E and F, tumor images in Fig. 7B and western blotting data shown in Fig. 4D had already appeared in previously published articles written by different authors at different research institutes (a few of which have been retracted). Owing to the fact that the contentious data in the above article had already been published prior to its submission to *Oncology Reports*, the Editor has decided that this paper should be retracted from the Journal. The authors were asked for an explanation to account for these concerns, but the Editorial Office did not receive a reply. The Editor apologizes to the readership for any inconvenience caused.

